# Unveiling bacterial communication with a MATLAB GUI implementing the diffusion-based quorum sensing model

**DOI:** 10.1038/s41598-024-63661-0

**Published:** 2024-06-07

**Authors:** Urvashi Singh, Zeeshan Saifi, Prem Saran Tirumalai, Soami Daya Krishnananda

**Affiliations:** 1 Department of Electrical Engineering, Indian Institute of Technology Bombay, Mumbai, India; 2 Department of Physics and Computer Science, Dayalbagh Educational Institute, Dayalbagh, Agra, Uttar Pradesh India; 3 Department of Agriculture Sciences (Botany), Dayalbagh Educational Institute, Dayalbagh, Agra, Uttar Pradesh India

**Keywords:** Quorum sensing, MATLAB, GUI, Organism and diffusion coefficient, Biological techniques, Biophysics, Computational biology and bioinformatics

## Abstract

Bacteria employ quorum sensing as a remarkable mechanism for coordinating behaviors and communicating within their communities. In this study, we introduce a MATLAB Graphical User Interface (GUI) that offers a versatile platform for exploring the dynamics of quorum sensing. Our computational framework allows for the assessment of quorum sensing, the investigation of parameter dependencies, and the prediction of minimum biofilm thickness required for its initiation. A pivotal observation from our simulations underscores the pivotal role of the diffusion coefficient in quorum sensing, surpassing the influence of bacterial cell dimensions. Varying the diffusion coefficient reveals significant fluctuations in autoinducer concentration, highlighting its centrality in shaping bacterial communication. Additionally, our GUI facilitates the prediction of the minimum biofilm thickness necessary to trigger quorum sensing, a parameter contingent on the diffusion coefficient. This feature provides valuable insights into spatial constraints governing quorum sensing initiation. The interplay between production rates and cell concentrations emerges as another critical facet of our study. We observe that higher production rates or cell concentrations expedite quorum sensing, underscoring the intricate relationship between cell communication and population dynamics in bacterial communities. While our simulations align with mathematical models reported in the literature, we acknowledge the complexity of living organisms, emphasizing the value of our GUI for standardizing results and facilitating early assessments of quorum sensing. This computational approach offers a window into the environmental conditions conducive to quorum sensing initiation, encompassing parameters such as the diffusion coefficient, cell concentration, and biofilm thickness. In conclusion, our MATLAB GUI serves as a versatile tool for understanding the diverse aspects of quorum sensing especially for non-biologists. The insights gained from this computational framework advance our understanding of bacterial communication, providing researchers with the means to explore diverse ecological contexts where quorum sensing plays a pivotal role.

## Introduction

Bacteria, though vastly different from humans in their evolutionary trajectory, exhibit remarkable social behavior and a profound capacity for communication with one another. This cellular interplay is orchestrated through a phenomenon known as quorum sensing^1^. Quorum sensing hinges on the release of diffusible signal molecules, termed autoinducers, by bacteria. Within this intricate signaling network, bacteria have developed the ability to govern gene expression in response to the density of autoinducers within their population, activating genetic pathways only when specific thresholds are reached^1^. This regulatory mechanism has evolved to impact a myriad of vital processes, encompassing biofilm formation, virulence, bioluminescence, motility, genetic competence, cooperative behaviors, and more^2^. The enigmatic world of quorum sensing, a fundamental communication mechanism employed by bacteria, has spurred an extensive exploration characterized by a diverse range of analytical methodologies and screening strategies. These techniques, including bioassays, thin-layer chromatography, mass spectrometry, gas chromatography, high-performance liquid chromatography, cell-based assays, and cell-free biosensors, have played pivotal roles in shedding light on this intricate phenomenon^3–6^.

While these methods have been instrumental in advancing our understanding of quorum sensing, their widespread utility has often been impeded by the formidable barriers of high cost. Such economic constraints have limited their accessibility, relegating the profound insights they offer to a select few. Furthermore, several of these approaches demand traditional culturing practices, which, by their nature, are inherently time-consuming. Additionally, the extensive use of reagents for preprocessing has further complicated their adoption^7^.

In response to these challenges, researchers have sought innovative ways that circumvent these limitations, envisioning a more accessible and efficient approach to unraveling the secrets of quorum sensing.

To address the complexity in the understanding of quorum sensing, researchers have proposed alternative methods that diverge from traditional empirical methods. Instead, they have turned to the development of theoretical mathematical models as indispensable tools for predicting various facets of quorum sensing dynamics^8–10^. In the context of this study. In this paper, we also propose a diffusion-based mathematical model meticulously translated into a user-friendly MATLAB Graphical User Interface (GUI).

The foremost objective of this research is to engage the community of microbiologists and researchers entrenched within the domain of quorum sensing. We aim to provide them with a robust computational tool encapsulated within the GUI, thus empowering them to make well-informed decisions during the initial phases of experimental design. Such a tool has the potential to not only expedite research efforts but also enhance the efficiency of subsequent laboratory investigations.Central to this approach is the fusion of mathematical modelling with user-friendly accessibility. Through this integration, we aspire to drive forward our comprehension of quorum sensing, contributing substantively to the overarching mission of unravelling the intricate tapestry of bacterial communication. This endeavour represents a novel pathway toward comprehending the inner workings of quorum sensing, the precision of mathematical modelling with the practicality of a GUI interface.

## Results

**Figure 1:**
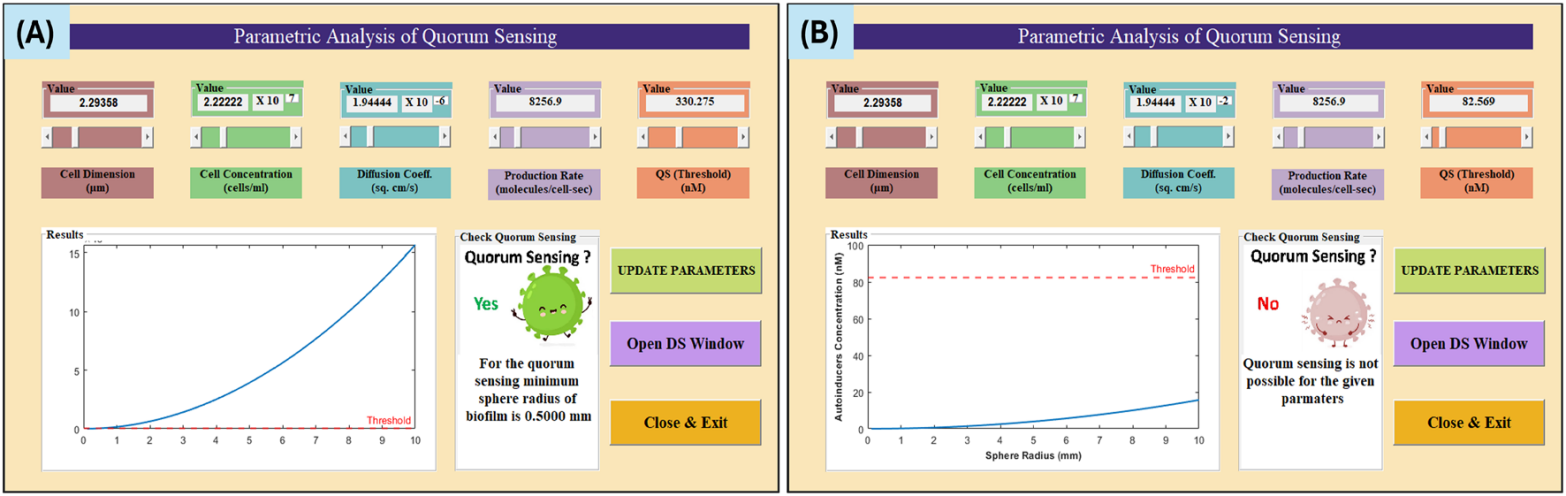
Example of the GUI result (**A**) when quorum sensing happens (screenshot) and (**B**) when quorum sensing does not happen (screenshot).
  The MATLAB Graphical User Interface (GUI) developed in this study offers several noteworthy features that aid in understanding quorum-sensing dynamics. These features enable the theoretical prediction of quorum sensing dependence on key parameters, including p (production rate of autoinducers), *n* (number of bacterial cells), *R* (radius of the bacterial cell cluster), *D* (diffusion coefficient of the medium), and $W_{s}$ (bacterial width). Importantly, the GUI takes into account the often-neglected factors of the error function and bacterial width, factors that are indeed crucial in the real-world scenario. Attempts to understand and predict the multi-variable nature of quorum sensing have always been through mathematical simplifications, which have sometimes omitted certain critical elements for the sake of simplicity. In the GUI-based mathematical model presented in this work, we have incorporated these often ignored key variables for holistic prediction. Therefore this work can also serve as an analytical tool to explore the subtle interplay of these parameters and their influence on quorum sensing outcomes with greater accuracy and completeness. In the subsequent sections, we studied the practical utility and implications of these features, highlighting their importance in advancing the predictive capabilities of our computational tool in analyzing the multi-variable phenomenon of bacterial quorum sensing.
- **Role of diffusion coefficient on quorum sensing** The diffusion coefficient is important in the evolutionary dynamics of quorum sensing. The diffusion mechanism serves as the mediator to convey the environmental conditions for bacterial communication. This parameter is inherently reliant on factors such as temperature and the viscosity of the medium^11^, thereby offering valuable insights into the environmental effects on quorum sensing systems. In particular, the diffusion coefficient within biofilms (referred to as $D_{biofilm}$) differs significantly from that in water ($D_{water}$) due to the presence of bacteria and extracellular polymer matrices within the biofilm. Empirical observations have revealed a reduction ratio ($D_{biofilm}$/$D_{water}$) typically ranging from 0.2 to 0.8^11^. Notably, the diffusion coefficient is not only temperature-dependent but also specific to different autoinducers. For instance, studies by Stewart et al.^12^ have reported diffusion coefficients of specific autoinducers (N-3 oxododecanoyl-L-HL and N-butyryl-HL) in water at room temperature^11^, underlining the autoinducer-specific nature of diffusion coefficients. Consequently, the diffusion coefficient can serve as a discriminatory parameter for distinguishing quorum sensing among various bacterial species, each of which diffuses distinct autoinducers.
  In our analysis, the production rate (expressed in autoinducer molecules per cell per second) is not held constant but is allowed to vary within the range of 500 to 50,000^13–16^. It is important to note that the maximum production rate (50,000) has been experimentally validated^13^. For the purposes of this study, a production rate of 5,000 is employed to explore the impact of diffusion coefficient variations.
  Considering the typical dimensions of a bacterial cell (approximately 1 µm) and an average bacterial cell concentration of $1\times 10^\mathrm{6}$/ml for quorum sensing, we set the biofilm thickness at 500 µm. In this context, we investigate quorum sensing behavior by systematically altering the diffusion coefficient within the range of $1\times 10^\mathrm{-6}$ cm^2^/sec to $1\times 10^\mathrm{-5}$ cm^2^/sec. The theoretical insights into the possibilities of quorum sensing can be gleaned from Fig. 2A. It becomes evident that quorum sensing is feasible within the range of diffusion coefficients from $1\times 10^\mathrm{-6}$ cm^2^/sec to $5 \times 10^\mathrm{-6}$ cm^2^/sec. Beyond this range, as the diffusion coefficient exceeds $6\times 10^\mathrm{-6}$ cm^2^/sec up to $1\times 10^\mathrm{-5}$ cm^2^/sec, the autoinducer concentration fails to reach the requisite threshold, rendering quorum sensing improbable. Intriguingly, as the diffusion coefficient increases, there is a concomitant decrease in autoinducer concentration. This observation implies that quorum sensing becomes more likely in thin bacterial films characterized by lower diffusion coefficients.

- **MATLAB GUI**
  Utilizing the mathematical model delineated (as discussed in the Methods Section), we have undertaken the development of a MATLAB Graphical User Interface (GUI). This GUI serves as a computational tool to forecast the likelihood of quorum sensing occurrence under specified conditions, characterized by the condition $N_{threshold} \le N_{total}$.
  GUI-based interface on providing the requisite parameter values, it furnishes two distinct outcomes, as illustrated in Fig. 1. These outcomes encapsulate critical insights into the feasibility of quorum sensing within the given context. The GUI represents a practical and user-friendly means of applying the mathematical model to real-world scenarios, thus facilitating informed decision-making in the study of bacterial communication and collective behaviours. In the subsequent sections, we elucidate the operation and significance of these outcomes, underscoring their importance in advancing our understanding of quorum sensing dynamics.

**Figure 2:**
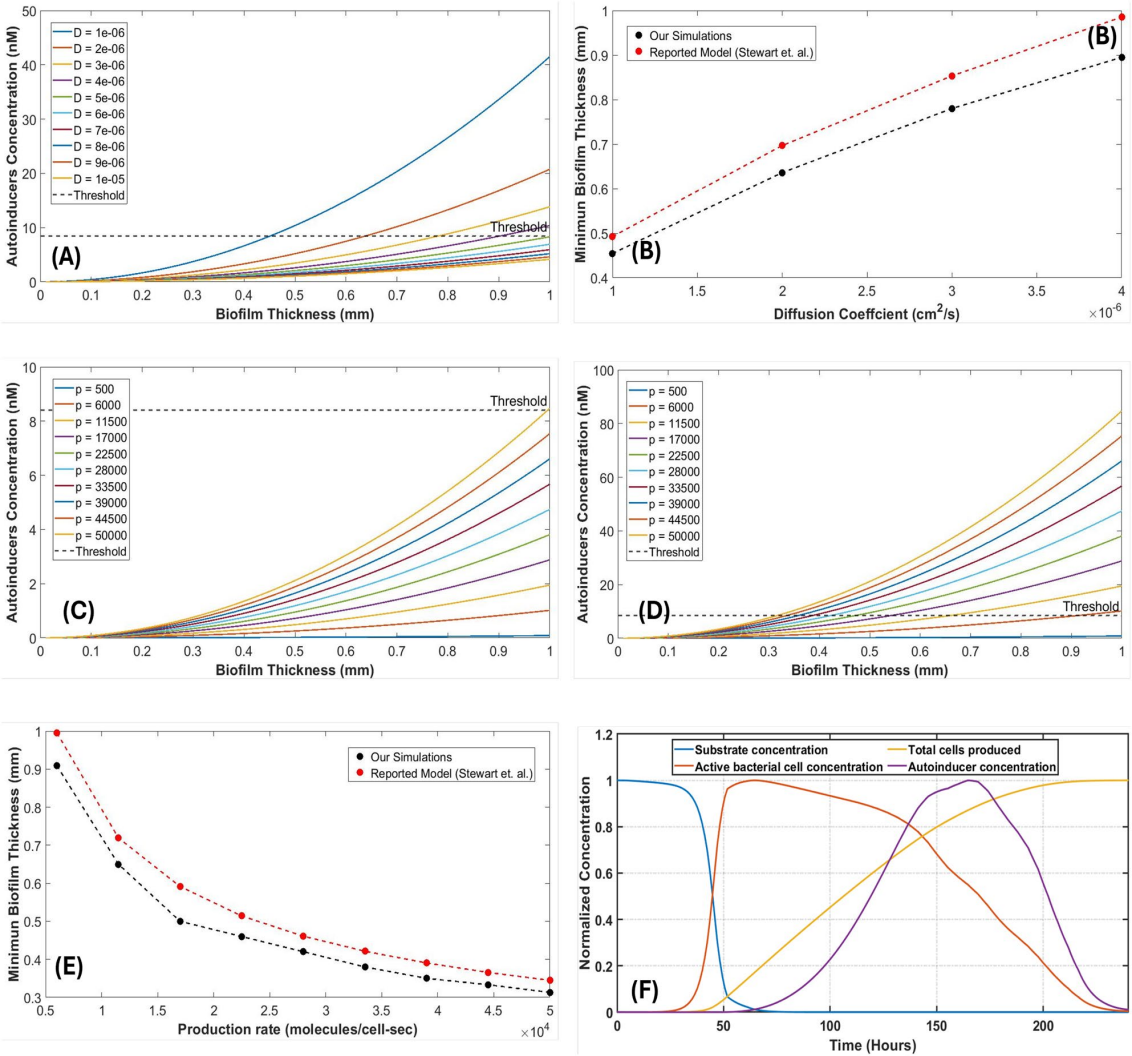
(**A**) Autoinducer concentrationas function of biofilm thickness (**B**) Minimum biofilm thickness required for triggering the quorum sensing mechanism for the particular diffusion coefficient. (**C**) Autoinducer concentration as a function of biofilm thickness and production rate for cell density 10^5^cells/ml and (**D**) for cell density 10^6^cells/ml (**E**) Minimum biofilm thickness required for triggering the quorum sensing mechanism for the production rate (**F**) Complete growth phase curve of microbial cells obtained through the Monod kinetic model encompassing the lag, log, stationery, the death phase along with cell concentration and autoinducer production Here P: production rate (autoinducer molecules/cell-sec) and D : diffusion coefficient cm^2^/sec.
  Furthermore, Fig. 2B offers insights into the minimum biofilm thickness required to trigger the quorum sensing mechanism. As quorum sensing occurs when the autoinducer concentration precisely reaches its threshold value, this minimum biofilm thickness ($Ws_{min}$) varies depending on the diffusion coefficient. For instance, at diffusion coefficients of $1\times 10^\mathrm{-6}$ cm^2^/sec, $2\times 10^\mathrm{-6}$ cm^2^/sec, $3\times 10^\mathrm{-6}$ cm^2^/sec, $4 \times 10^\mathrm{-6}$ cm^2^/sec, and $5\times 10^\mathrm{-6}$ cm^2^/sec, the corresponding minimum biofilm thicknesses are 0.45 mm, 0.62 mm, 0.78 mm, 0.88 mm, and 1 mm, respectively. This relationship aligns with findings reported by Stewart et al.^12^.
  In the subsequent sections, we delve deeper into the implications of these findings, shedding light on the intricate interplay between diffusion coefficients and the quorum sensing phenomenon, with potential applications in understanding and controlling bacterial communication.
- ** Role of production rate and cell concentration on quorum sensing** The production rate in quorum sensing corresponds to the rate at which bacterial cells release signaling molecules, known as autoinducers, per cell per second. These autoinducers accumulate in the surrounding environment and their density becomes directly proportional to the cell density. Quorum sensing, a phenomenon essential for bacterial coordination, is achieved when the autoinducer density surpasses a certain critical threshold.
  In our investigation, simulations were conducted for two distinct cell concentrations: 10^5^ cells/ml and 10^6^ cells/ml, as illustrated in Fig. 2C,D, respectively. It was observed that at a cell concentration of 10 ^5^ cells/ml, the autoinducer concentration reached the critical threshold only when the production rate was set at 50,000. This finding offers a compelling insight: “Two quorum systems featuring the same cell concentration but differing in production rates suggest that quorum sensing is attained earlier in the system characterized by a higher production rate.” Conversely, at a cell concentration of 10^6^ cells/ml, quorum sensing was attainable for all tested production rates except when set at 500 or below. Notably, as illustrated in Fig. 2E, the minimum biofilm thickness required for quorum sensing varied with different production rates. This underscores a nuanced relationship between production rates and the critical biofilm thickness at which quorum sensing becomes possible.
  Furthermore, intriguingly, varying the dimensions of the bacterial cells did not yield significant alterations in autoinducer concentration. This observation suggests that autoinducer density remains relatively stable across different cell dimensions, highlighting the prominence of production rates and cell concentrations as primary determinants of quorum sensing dynamics. In the ensuing sections, we delve into a comprehensive analysis of these findings, exploring the intricate interplay between production rates, cell concentrations, and biofilm dimensions in the context of quorum sensing. These insights are instrumental in elucidating the mechanisms governing bacterial coordination and communication within various ecological niches.
- ** Role of time-dependent microbial growth dynamics on autoinducer production** In addition to the factors like production rate, diffusion coefficient, and cell dimensions, it is also crucial to account for the effect of the time-dependent growth pattern of bacterial cells in a given medium (substrate) on quorum sensing. Since the autoinducer concentration explicitly depends on bacterial cell density, the dynamics of the substrate consumption by bacterial cells (to further yield cell division) must be considered for the time-dependent study of quorum sensing. Ultimately, which phase of the growth curve the microbial cells are in, also plays a significant role in the secretion of autoinducers. A single-species chemostat-kinetic type mathematical model has been utilized in the current study of time dynamics in quorum-sensing. Based on the classical Monod kinetic model^17^, which as per literature is a well-accepted and experimentally verified (ordinary differential equation) ODE-based model^18,19^, we calculated the bacterial cell density as a function of time. This time-dependent cell density, its associated production rate, and the incremental size of the volume sphere as a result of bacterial multiplication were used in the quorum sensing model to further comprehend the time dynamics of the system. Notably, the autoinducer secretion is expressed with a significant delay in contrast to the growth curve of bacteria, with the maxima in autoinducer concentration observed at the onset of the death phase which gradually declines due to the reduction in the number of active cells during nutrient-deprived condition.
  A complete growth phase curve of microbial cells obtained through the Monod kinetic model^17,18^ encompassing the lag, log, stationery, the death phase is shown in Fig. 2F for the time span of 240 hours. Detailed specifications of the model and the value of parameters used are given in the Methods section. The concentrations plotted in Fig. 2F were normalized for a comparative analysis between the microbial cell concentration and autoinducer production. It is evident from the figure that in addition to the diffusive geometric expansion of the colony, which is primarily due to the increased cell population, the availability of growth nutrients and the life-span of microbial cells are also important while studying the collective behavior of microbes such as quorum sensing. The trend obtained for the time-dependent analysis of substrate utilization and biomass production (cell multiplication) in our study matches well with the results reported by Kim et al.^19^ for mono-cultures and Gude et al.^20^ for co-cultures both numerically and experimentally through optical density (OD) measurements.
  Since the time dynamics of such a phenomenon depends on quite a large number of parameters and during the numerical analysis of the theoretical model we are constrained to keep the value of many of these parameters fixed while varying a few. This is often practiced to properly visualize the effect of the concerned parameters on the dynamic systems. Thus, an associated GUI for studying the time dynamics with the flexibility to vary a large number of parameters is additionally incorporated. This subsidiary GUI (as shown in Fig. 3) focuses on the time-dependent analysis of microbial growth and autoinducer concentration rather than forecasting the likelihood of quorum sensing occurrence which has already been dealt with in the previous sections. Such studies are central to not only the analysis of the formation, maturity, and dispersal of biofilms through the quorum sensing mechanism but are also helpful in the study of bio-remediation and efficacy of antimicrobial complexes^21^.

**Figure 3:**
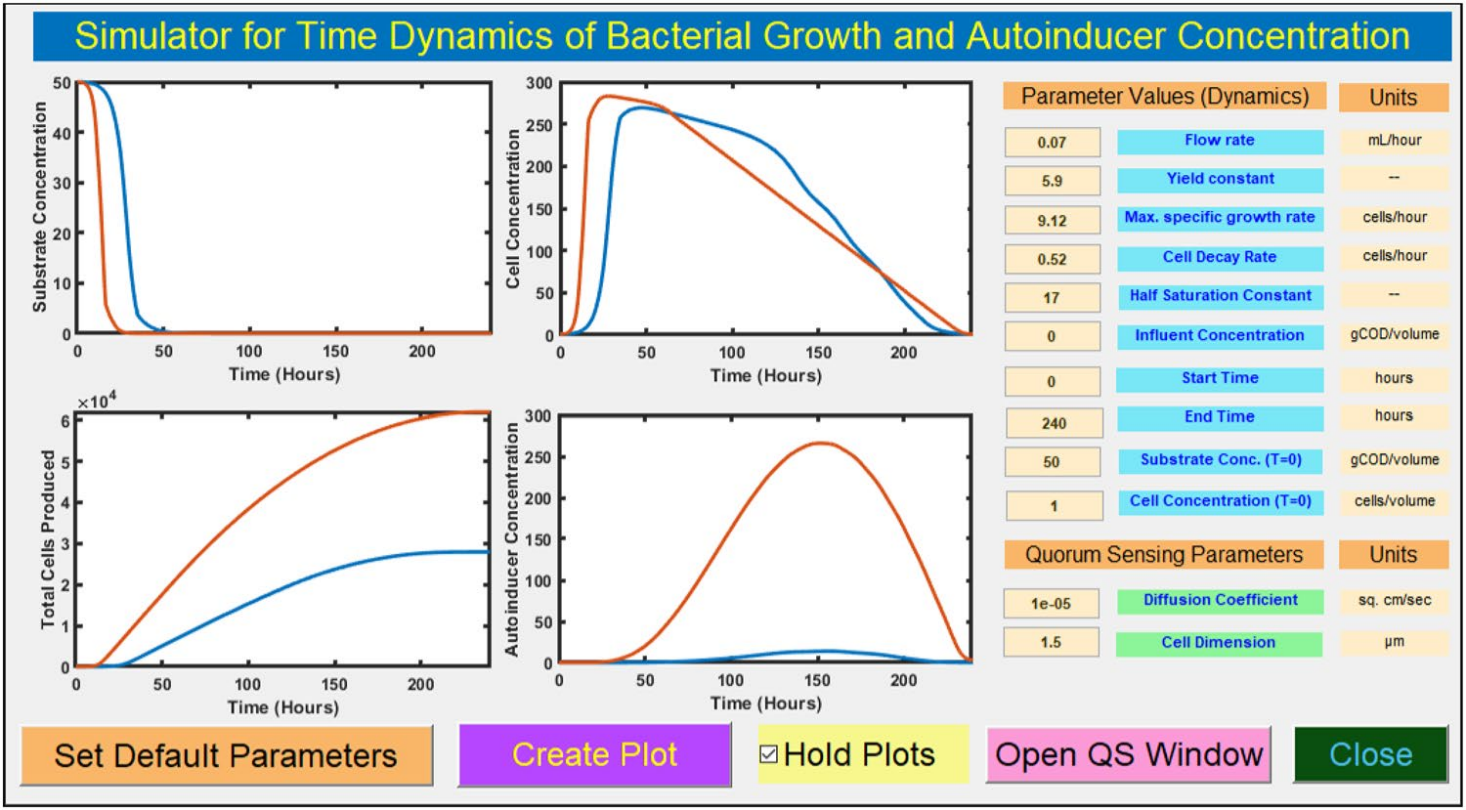
Graphical user interface of the simulator design to study the time-dependent variation in autoinducer production based on the microbial growth phase. The red and blue curves plotted in each subplot window indicate the change in the concerned variables like substrate and cell concentration and the associated autoinducer production as a function of time for the value of half-saturation constant as $K_{s}=55.2$ for blue curve and $K_{s}=17$ for the red curve, all other parameters were kept at the values indicated in the figure except $K_{s}$. (The dependence of the other parameters can be visualized through the GUI appended as the Supplementary File).

## Discussion

The MATLAB Graphical User Interface (GUI) developed in this study stands as a versatile tool with multiple capabilities, including the assessment of quorum sensing, exploration of parameter dependencies, and the determination of the minimum biofilm thickness necessary to initiate quorum sensing. Our simulations have yielded several salient insights into the mechanisms governing quorum sensing, which have significant implications for understanding bacterial coordination and communication in various ecological contexts. One of the key findings underscores the paramount importance of the diffusion coefficient in quorum sensing compared to bacterial cell dimensions. By systematically varying the diffusion coefficient over the range of 1$\times$10^-6^ cm^2^/sec to $1\times 10^\mathrm{-5}$ cm^2^/sec, we observed substantial fluctuations in autoinducer concentration. Notably, this pronounced variability was not observed when altering cell dimensions from 1 $\mu$m to 10 $\mu$m. This insight underscores the central role of the diffusion coefficient as a primary determinant of quorum sensing dynamics, emphasizing its significance in shaping the communication capabilities of bacterial populations.

Furthermore, our GUI allows for the prediction of the minimum biofilm thickness required to trigger quorum sensing, which is contingent on the diffusion coefficient. This feature is particularly valuable for understanding scenarios where biofilms exhibit limited thickness, corresponding to lower diffusion coefficients. The capability to estimate this critical biofilm thickness provides essential insights into the spatial constraints governing quorum sensing initiation, offering a valuable tool for researchers exploring biofilm-associated phenomena.

Our simulations shed light on the interplay between production rates, cell concentrations, and quorum sensing. We observed that varying the production rate while maintaining the same cell concentration resulted in earlier quorum sensing initiation when the production rate was higher. Conversely, keeping the production rate constant while altering cell concentrations revealed a similar trend, with higher cell concentrations leading to earlier quorum sensing attainment. These observations underscore the intricate relationship between cell communication and the population dynamics of bacterial communities, indicating that higher production rates or cell concentrations expedite quorum sensing. While our simulations align with mathematical models reported in the literature, it is crucial to acknowledge that bacteria are living organisms, characterized by intricate and adaptive behaviours that extend beyond the scope of theoretical modelling^20,21^. Consequently, this GUI serves as a valuable resource for microbiologists seeking to standardize their results and predictions, facilitating early assessments of quorum sensing. Such studies have the potential to provide insights into the environmental conditions favourable for quorum sensing initiation, encompassing parameters such as diffusion coefficient, cell concentration, and biofilm thickness.

In summary, the flexibility and capabilities of our MATLAB GUI offer a valuable tool for comprehending the multifaceted world of quorum sensing. The insights gained from this computational approach advance our understanding of bacterial communication, enabling researchers to explore the diverse ecological contexts where quorum sensing plays a pivotal role.

## Conclusion

In this study, we introduced a versatile MATLAB Graphical User Interface (GUI) for exploring bacterial quorum sensing dynamics. Our simulations revealed that the diffusion coefficient plays a pivotal role, surpassing bacterial cell dimensions in influencing quorum sensing. Predictive capabilities of our GUI shed light on the minimum biofilm thickness needed for quorum sensing initiation. We also highlighted the interplay between production rates and cell concentrations in quorum sensing, showing that higher rates or concentrations expedite this phenomenon. While our simulations align with existing models, we emphasized the GUI’s utility for standardizing results and facilitating early assessments of quorum sensing in biofilms.

Biofilms have the potential to be manipulated to enhance the expression of particular metabolic pathways, hence leading to an enhancement in the synthesis of desired chemicals. This discipline of biofilm engineering is characterized by its dynamic nature and continuous evolution since it aims to exploit the distinctive attributes of biofilms for many practical purposes. The subject matter encompasses a comprehension of the processes involved in the creation, structure, and function of biofilms, as well as the advancement of novel methodologies aimed at regulating and customizing biofilm characteristics to meet specific requirements. This is pertinent to the concepts of thickness and quorum sensing. The determination of the structure and functioning of the microbial community within a biofilm has been found to be influenced by its thickness. Moreover, the initiation of quorum sensing is contingent upon the presence of a minimum biofilm thickness, which subsequently influences the regulation of certain gene expressions mediated by quorum sensing. The expression of these genes may play a significant role in shaping the characteristics of the microbial community. Our model in that sense also raises the question of whether quantifying quorum sensing could serve as an indicator for biofilm thickness, production rates, and cell concentrations.

In summary, our MATLAB GUI provides an invaluable tool for understanding quorum sensing and biofilms, advancing our knowledge of bacterial communication, and enabling exploration of its diverse ecological contexts. By integrating time-dependent microbial growth dynamics and autoinducer production, our research sheds light on the intricate relationship between bacterial behavior and environmental factors, offering insights crucial for biofilm formation, bio-remediation strategies, and optimizing antimicrobial efficacy.

## Methods

Understanding the intricate dynamics of quorum sensing in bacterial populations is central to deciphering their coordinated behaviors. Bacteria employ autoinducers, diffusible signalling molecules, as a means of communication within their growth environment. The theoretical foundation for quorum sensing can be established through the application of Fick’s law of diffusion theory^13^. Consider a scenario where bacterial cells release autoinducers into the environment with a time-independent production rate. The concentration of these autoinducers (N) at a given position (r) and time (t) can be described using Fick’s second law^13^.

1 $$\begin{aligned} \frac{\partial N(r,t)}{\partial t} =D\nabla ^{2} \ N(r,t)+ P\ \frac{e^{-r^2/2W_s}}{(2\pi W_s^2)^{3/2}}\ \end{aligned}$$

Here, D represents the diffusion coefficient of the medium, and P denotes the production rate of autoinducers. The solution to this equation can be determined through a mathematical approach developed by Pikulin et al.^12^, yielding:

2 $$\begin{aligned} N(r,t) =\frac{P}{2 \pi Dr} \phi \left( \frac{r}{\sqrt{2} W_s}\right) \end{aligned}$$

Where $\phi$ (the error function) can be expressed in integral form as:

3 $$\begin{aligned} \phi (x) = \frac{2}{\sqrt{\pi }} \int _0^x e^{-h^2} dh \end{aligned}$$

This function is monotonically increasing, approaching 1 as r tends to infinity. Consequently, at r=$\infty$, the concentration can be approximated as:

4 $$\begin{aligned} N(r=\infty ) = \frac{P}{2 \pi Dr} \end{aligned}$$

Using the Taylor expansion, at r=0, the above Eq. (2) can be solved using the Taylor approximation.

5 $$\begin{aligned} N(r=0) = \frac{1}{(2\pi )^{3/2}} \frac{P}{DW_s} \end{aligned}$$

To extend this formulation to n bacterial cells, Eq. (1) can be expressed as:

6 $$\begin{aligned} \frac{\partial N(r,t)}{\partial t} = D\ \nabla ^{2} \ N(r,t)+ \sum _{j=1}^{n} p\ \frac{e^{-(r-r_j)^2/2W_s}}{(2\pi W_s^2)^{3/2}} \end{aligned}$$

Similarly, the time-independent solution at the origin (r=0) can be derived as:

7 $$\begin{aligned} N_{total}(r=0) =\frac{Pn}{4 \pi D} \int _0^R 4\pi r^2 \frac{1}{r} \phi \left( \frac{r}{\sqrt{2} W_s}\right) \end{aligned}$$

This equation can be further refined using Taylor series^13^:

8 $$\begin{aligned} N_{total}(r=0)=\frac{Pn R^2}{2D} \left[ 1-\frac{W_s^2}{R^2} \phi \left( \frac{R}{\sqrt{2} W_s}\right) +\sqrt{\frac{2W_s}{\pi R} e^{-(R/2W_s)^2}} \right] \end{aligned}$$

In addition to the diffusion and density-mediated spatial dependence of the autoinducer concentration for the study of quorum sensing, aspects of the interplay between the cell viability (based on the different temporal phases of the microbial growth) and secretion of autoinducers as a function of time should not be overlooked. A small number of microbial cells inoculated in bulk of substrate (source of nutrients) passes through different phases, each having a different population of active cells which is dependent on the rate at which the microbes digest the substrate, as well as, on the rate at which the microbial cells multiply. In our case of single species colonies, the most general form of the equations that provide a local solution for such bioreactor type of a system is given by the following equations^17,18^:

9 $$\begin{aligned} \frac{d\theta }{dt}= & {} (\theta _{in} - \theta ) \alpha -(\mu (\theta ) \beta ) \end{aligned}$$

10 $$\begin{aligned} \frac{d\beta }{dt}= & {} (\gamma \mu (\theta ) - \alpha )\beta -k_{dec}\beta \end{aligned}$$

Where, using Monod model, $\mu (\theta )$ can be expressed as:

11 $$\begin{aligned} \mu (\theta )=\frac{\mu _{max}\theta }{K_{s}+\theta } \end{aligned}$$

To study the time dynamics of autoinducer concentration, (*n*) is Eq. (8) was replaced by a time-dependent ODE solution of $(\beta )$ in Eqs. (9), (10), and (11). This value $(\beta )$ as a function of time was further used to calculate, the radius of the sphere encompassing these cells (*R*), and the production rate (*P*) without varying other parameters of Eq. (8). Specifications of all the parameters used in method section are shown in Tables 1 and 2.

**Table 1:** Specification of the parameters used time-independent analysis.

Parameter	Specification	Units
*P*	Production rate	*molecules per cell* $\textrm{sec}^{-1}$
*n*	Cell density	*cell*/*ml*
*N*	Autoinducer concentration	*nM*
*R*	Radius of cell cluster	*mm*
*D*	Diffusion coefficient	$cm^{2}\textrm{sec}^{-1}$
$W_{s}$	Bacterial cell width	$\mu$m
*t*	Time	*seconds*
*r*	Spatial position	*mm*

**Table 2:** Specification of the parameters used in time-dependent analysis.

Parameter	Specification	Units
$\theta$	Substrate concentration	*gCOD*/*mL*
$\beta$	Cell density	*no*. *of* *cells*/*mL*
$\theta _{in}$	Influent concentration	*gCOD*/*mL*
$\alpha$	Flow rate	*mL*/*hour*
$\mu$	Microbial growth rate	*no*. *ofcells*/*hour*
$\gamma$	Biomass yield constant	–
$K_{s}$	Half saturation constant	−
$k_{dec}$	Cell decay rate	*no*. *of* *cells*/*hour*
$\mu _{max}$	Maximum specific growth rate	*no*. *of* *cells*/*hour*
*t*	Time	*Hours*

Quorum sensing is said to occur when the total concentration of autoinducers $(N_{total})$ surpasses a threshold value $(N_{threshold})$. Here, we consider a spherical coordinate system, with R representing the radius of the sphere encompassing all bacterial cells. In the subsequent sections, we investigate the implications and applications of this mathematical framework for quorum sensing, elucidating the critical role it plays in understanding bacterial communication and collective behaviors.

### Supplementary Information

Supplementary Information.

## Data Availability

The datasets and codes used and/or analyzed during the current study are available from the corresponding author (S.D.K) on reasonable request.
