# Supplementary figures and images for: Unveiling bacterial communication with a MATLAB GUI implementing the diffusion-based quorum sensing model
Source: Sci Rep. 2024 Jun 7;14:13104. doi: 10.1038/s41598-024-63661-0 (PMC11161612; doi:10.1038/s41598-024-63661-0)

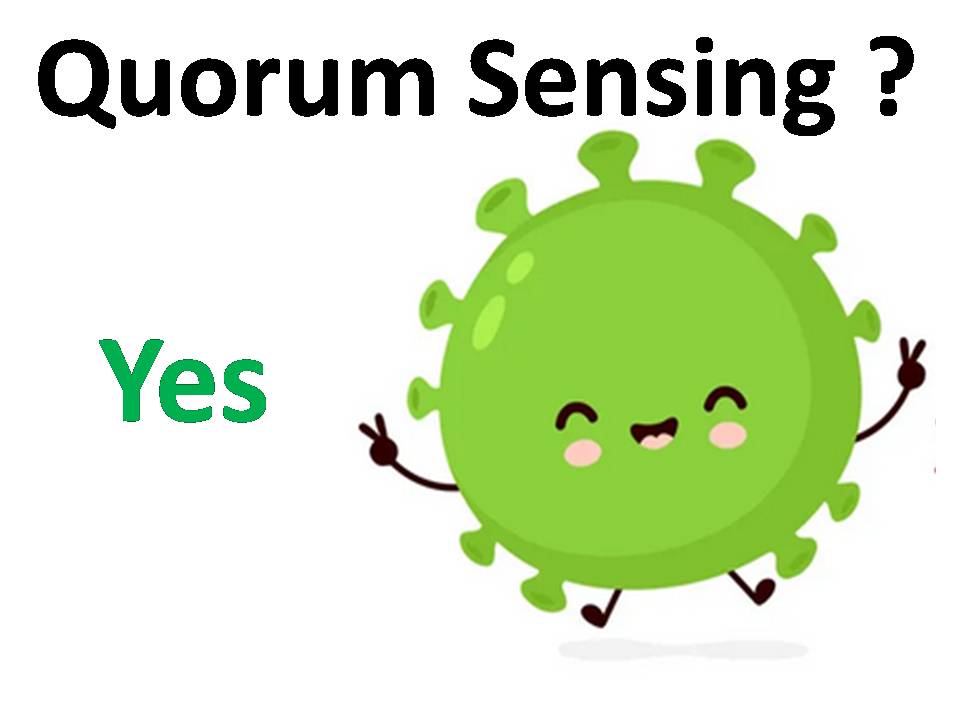

Supplement: Supplementary file 1 — Supplementary Information. [file 41598_2024_63661_MOESM1_ESM.zip › Slide1.JPG]

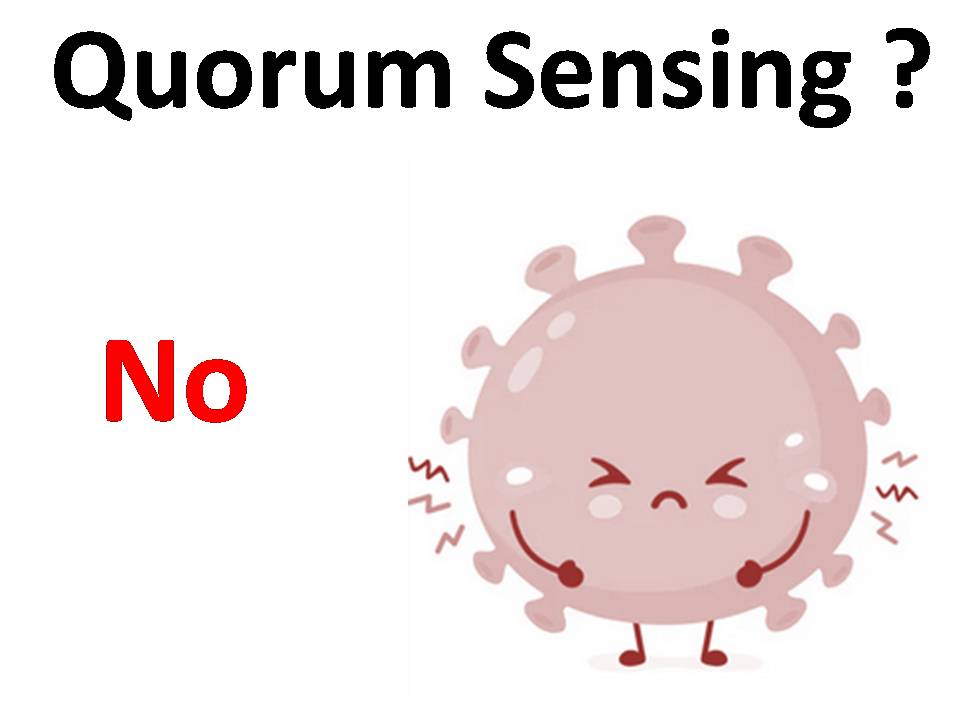

Supplement: Supplementary file 1 — Supplementary Information. [file 41598_2024_63661_MOESM1_ESM.zip › Slide2.JPG]
